# qKAT: Quantitative Semi-automated Typing of Killer-cell Immunoglobulin-like Receptor Genes

**DOI:** 10.3791/58646

**Published:** 2019-03-06

**Authors:** Jyothi Jayaraman, Vitalina Kirgizova, Da Di, Christopher Johnson, Wei Jiang, James A. Traherne

**Affiliations:** 1 Department of Pathology, University of Cambridge; 2 Department of Physiology, Development and Neuroscience, University of Cambridge; 3 Department of Obstetrics and Gynaecology, University of Cambridge School of Medicine, NIHR Cambridge Biomedical Research Centre; 4 Centre for Trophoblast Research, University of Cambridge; 5 Department of Genetics & Evolution, University of Geneva; 6 Royal Papworth Hospital; 7 Department of Plant Sciences, University of Cambridge

**Keywords:** Genetics, Issue 145, Immunogenetics, killer cell immunoglobulin-like receptor (KIRs), copy number variation, haplotype, human leukocyte antigen (HLA), natural killer cell, quantitative polymerase chain reaction (qPCR)

## Abstract

Killer cell immunoglobulin-like receptors (KIRs) are a set of inhibitory and activating immune receptors, on natural killer (NK) and T cells, encoded by a polymorphic cluster of genes on chromosome 19. Their best-characterized ligands are the human leukocyte antigen (HLA) molecules that are encoded within the major histocompatibility complex (MHC) locus on chromosome 6. There is substantial evidence that they play a significant role in immunity, reproduction, and transplantation, making it crucial to have techniques that can accurately genotype them. However, high-sequence homology, as well as allelic and copy number variation, make it difficult to design methods that can accurately and efficiently genotype all *KIR* genes. Traditional methods are usually limited in the resolution of data obtained, throughput, cost-effectiveness, and the time taken for setting up and running the experiments. We describe a method called quantitative *KIR* semi-automated typing (qKAT), which is a high-throughput multiplex real-time polymerase chain reaction method that can determine the gene copy numbers for all genes in the *KIR* locus. qKAT is a simple high-throughput method that can provide high-resolution *KIR* copy number data, which can be further used to infer the variations in the structurally polymorphic haplotypes that encompass them. This copy number and haplotype data can be beneficial for studies on large-scale disease associations, population genetics, as well as investigations on expression and functional interactions between *KIR* and *HLA*.

## Introduction

In humans, the killer immunoglobulin-like receptor*(KIR*) locus is mapped on the long arm of chromosome 19 within the leukocyte receptor complex (LRC). This locus is around 150 kb in length and includes 15 *KIR* genes arranged head-to-tail. The *KIR* loci that are currently known are *KIR2DL1*, *KIR2DL2*/*KIR2DL3*, *KIR2DL4*, *KIR2DL5A*, *KIR2DL5B*, *KIR2DS1-5*, *KIR3DL1*/*KIR3DS1*, *KIR3DL2-3*, and two pseudogenes, *KIR2DP1* and *KIR3DP1*. The *KIR* genes encode for two-dimensional (2D) and three-dimensional (3D) immunoglobulin-like domain receptors with short (S; activating) or long (L; inhibitory) cytoplasmic tails, which are expressed by natural killer (NK) cells and subsets of T cells. Copy number variation exhibited within the *KIR* locus forms diverse haplotypes with variable gene content^1^. Non-allelic homologous recombination (NAHR), facilitated by a close head-to-tail gene arrangement and high-sequence homology, is the mechanism proposed to be responsible for the haplotypic variability. Over 100 different haplotypes have been reported in populations worldwide^1,2,3,4^. All these haplotypes could be divided into two major groups: A and B haplotypes. The A haplotype contains 7 *KIR* genes: *KIR3DL3*, *KIR2DL1*, *KIR2DL3*, *KIR2DL4*, *KIR3DL1*, and *KIR3DL2*, which are inhibitory *KIR* genes, and the activating *KIR* gene *KIR2DS4*. However, up to 70% of European-origin individuals who are homozygous for KIR haplotype A exclusively carry a non-functional "deletion" form of *KIR2DS4*^5,6^. All other *KIR* gene combinations form group B haplotypes, including at least one of the specific *KIR* genes *KIR2DS1*, *KIR2DS2*, *KIR2DS3*, *KIR2DS5*, *KIR3DS1*, *KIR2DL2*, and *KIR2DL5*, and typically include two or more activating *KIR* genes.

HLA Class I molecules have been identified as the ligands for certain inhibitory receptors (*KIR2DL1*, *KIR2DL2*, *KIR2DL3*, and *KIR3DL1*), activating receptors (*KIR2DS1*, *KIR2DS2*, *KIR2DS4*, *KIR2DS5*, and *KIR3DS1*), and for *KIR2DL4*, which is a unique *KIR* that contains a long cytoplasmic tails like other inhibitory KIR receptors but also has a positively charged residue near the extracellular domain which is a common feature of other activating *KIR* receptors. The combination of variants within the KIR genes and the HLA genes influences receptor ligand interaction that shapes potential NK cell responsiveness at the individual level^7,8^. Evidence from genetic association studies has indicated that *KIR* plays a role in viral resistance (e.g., human immunodeficiency virus [HIV]^9^ and hepatitis C virus [HCV]^10^), the success of transplantation^11^, the risk of pregnancy disorders and reproductive success^12,13^, the protection against relapse after allogeneic hematopoietic stem cell transplantation (HSCT)^14,15,16^, and the risk of cancers^17^.

The combination of high-sequence homology and allelic and haplotypic diversity presents challenges in the task of accurately genotyping *KIR* genes. Conventional methods to type *KIR* genes include sequence-specific primer (SSP) polymerase chain reaction (PCR)^18,19,20^, sequence-specific oligonucleotide probe (SSOP) PCR^21^, and matrix assisted laser desorption ionization-time of flight mass spectrometry (MALDI-TOF MS)^22^. The drawbacks of these techniques are that they only provide partial insight into the genotype of an individual whilst also being laborious to perform. Recently next-generation sequencing (NGS) has been applied to type the *KIR* locus specifically. While this method is very powerful, it can be expensive to run, and it is time-consuming to conduct in-depth analysis and data checks.

qKAT is a high-throughput quantitative PCR method. While conventional methods are laborious and time-consuming, this method makes it possible to run nearly 1,000 genomic DNA (gDNA) samples in five days and gives the *KIR* genotype, as well as the gene copy number. qKAT consists of ten multiplex reactions, each of which targets two *KIR* loci and one reference gene of a fixed copy number in the genome (*STAT6*) used for the relative quantification of the *KIR* gene copy number^23^. This assay has been successfully used in studies involving large population panels and disease cohorts on infectious diseases such as HCV, autoimmune conditions like type 1 diabetes, and pregnancy disorders such as preeclampsia, as well as providing a genetic underpinning to studies aimed at understanding the NK cell function^1,4,24,25,26^.

## Protocol

### 1 Preparation and Plating out of DNA

1. Accurately quantify the gDNA concentration using a spectrophotometric or fluorometric instrument.
2. Dilute DNA to 4 ng/μL on a 96-well deep-well plate. Include at least one control gDNA sample with a known copy number and one non-template control.
3. Centrifuge the 96-well plates at 450 x *g* for 2 min.
4. Using a liquid handling instrument, dispense each sample in quadruplicate onto 384-well qPCR plates so that every well has 10 ng of DNA (2.5 μL/well). Prepare at least ten 384-well plates, one for each qKAT reaction.
5. If gDNA is being dispensed from more than one 96-well plate, perform a full-volume wash with 2% bleach and ultrapure water to clean the needles of the liquid handling system between each 96-well plate of gDNA samples.
6. Air-dry the DNA by incubating the 384-well plates in a clean area at room temperature for at least 24 h.

### 2 Preparation of the Primers and Probes

**NOTE:** qKAT consists of ten multiplex reactions. Each reaction includes three primer pairs and three fluorescence-labeled probes that specifically amplify two *KIR* genes and one reference gene. The probes that were published in Jiang et al.^27^ were modified so that the oligonucleotides are now labeled with ATTO dyes since they offer improved photostability and long signal lifetimes. Pre-aliquoted primer combinations are commercially available (see **Table of Materials**).

1. Prepare primer combinations for each reaction as per the dilutions given in Table 1.
2. Prepare probe combinations for each reaction as per Table 1. Test each individual probe prior to making the combination.

### 3 Preparation of the Master Mix

**NOTE** The volumes mentioned below are for performing one qKAT reaction on a set of 10x 384-well plates.

1. Ensure that the gDNA samples plated on the 384-well plates are completely dry. Conduct all steps on ice and keep the reagents covered from exposure to light as much as possible since the fluorescence-labeled probes are photo- and thermo-sensitive.
2. Defrost the qPCR buffer, primer, and probe aliquots at 4 °C.
3. On ice, prepare a master mix for 10x 384-well plates by adding 18.86 mL of ultrapure water, 20 mL of qPCR buffer, 1,000 μL of preprepared primer combination, and 180 μL of preprepared probe combination (Table 2).
4. Distribute the master mix evenly across a 96-deep well plate using a multi-channel pipette, pipetting 415 μL into each well. Keep this plate in an ice box covered from light.
5. Using a liquid handling instrument, dispense 9.5 μL of the master mix into each well of the 384-well plate with dried gDNA. Seal the plate with a foil and immediately place it at 4 °C. Repeat this process for the remaining plates, ensuring that the needles of the liquid handling system are washed with water between each plate.
6. Centrifuge the 384-well plates at 450 x g for 3 min and incubate them at 4 °C overnight or between 6 - 12 h to resuspend the DNA and to dissipate any air bubbles.

### 4 qPCR Assay

1. Following the overnight incubation, centrifuge at 450 x *g* for 3 min to dissipate any remaining air bubbles.
2. For purposes of automation, connect the qPCR machine (e.g., LightCycler 480) to a microplate handler (see **Table of Materials**). Program the microplate handler to place the plates into the qPCR machine from a cooled storage dock that is protected from light.
  **NOTE** The assays should, in theory, work on other qPCR machines with compatible optic settings.
3. Use the following cycling conditions: 95 °C for 5 min followed by 40 cycles of 95 °C for 15 s and 66 °C for 50 s, with data collection at 66 °C.
4. Once the run is complete, have the robot collect the plate from the qPCR machine and place it in the discard dock.

### 5 Post-run Analysis

1. After amplification, calculate the quantification cycle (Cq) values using either the second derivative maximum method or the Fit Points method with the software of the qPCR machine (see **Table of Materials**), following the steps below.
2. Open the qPCR software and, in the **Navigator** tab, open the saved reaction experiment file for one plate.
3. **For the analysis using the second derivative maximum method, select the Analysis tab, and create a new analysis using Abs Quant/Second Derivative Max method**.
  1. In the **Create new analysis** window, select analysis type: **Abs Quant/Second Derivative Max method**, subset: **All Samples**, program: **Amplification**, name: **Rx-DFO** (where **x** is the reaction number).
  2. Select **Filter Comb** and choose **VIC/HEX/Yellow555 (533-580)**. This ensures that the data collected for *STAT6* is selected.
  3. Select **Colour Compensation** for **VIC/HEX/Yellow555(533-580)**. Click **Calculate**. Repeat this for Fam (465-510) and Cy5/Cy5.5(618-660). Click **Save file**.
4. **For the analysis using the Fit Points method, select Abs Quant/Fit Points in the Analysis tab**.
  1. In the **Create new analysis**window, select analysis type: **Abs Quant/Fit Points method**, subset: **All Samples**, program: **Amplification**, name: **RxF-DFO**(where **x** is the reaction number).
  2. Select the correct filters and color compensations for *STAT6* and each of the *KIR* genes (Fam/Cy5). In the **Noiseband** tab, set the noise band to exclude the background noise.
  3. In the **Analysis** tab, set the fit points to **3** and select **Show fit points**. Click **Calculate**. Click **Save file**.

### 6 Export of the Results

1. In the qPCR software, open the **Navigator** tab. Select **Results Batch Export**.
2. Open the folder in which the experiment files are saved and transfer the files into the right-hand side section of the window. Click **Next**. Select the name and the location of the export file.
3. Select Analysis type **Abs Quant/Second Derivative Max method** or **Abs Quant/Fit Points**. Click **Next**. Check that the name of the file, the export folder, and the analysis type are correct and click **Next** to start the export process.
4. Wait until the **Export Status** is **Ok**. The screen will automatically move to the next step. Check that all selected files have been exported successfully so that the number of files failed = 0. Click **Done**.
5. Use scripts split_file.pl and roche2sds.pl to split the exported plates into individual reactions for each plate.
  **NOTE** The scripts are provided on request/GitHub.

### 7 Copy Number Calculations

1. Open the copy number analysis software (e.g., CopyCaller). Select **Import real-time PCR results file** and load text files created by **roche2sds.pl**.
2. Select **Analyze** and conduct the analysis by either selecting **calibrator sample with known copy number** or by selecting **most frequent copy number**. See Table 5 for the most frequent copy number of *KIR* genes typically observed in European-origin populations.

### 8 Data-quality Checks

1. Use R script **KIR_CNVdata_analysis_for_Excel_ver020215.R** to combine copy number data from all the plates into a spreadsheet.
  **NOTE** The scripts are provided on request/GitHub.
2. Recheck the raw data on the copy number analysis software for samples that do not conform to the known linkage disequilibrium (LD) for *KIR* genes (Table 6).

## Representative Results

Copy number analysis can be carried out by exporting the files to the copy number analysis software, which provides the predicted and estimated copy number based on the ΔΔCq method.

**Figure 1:**
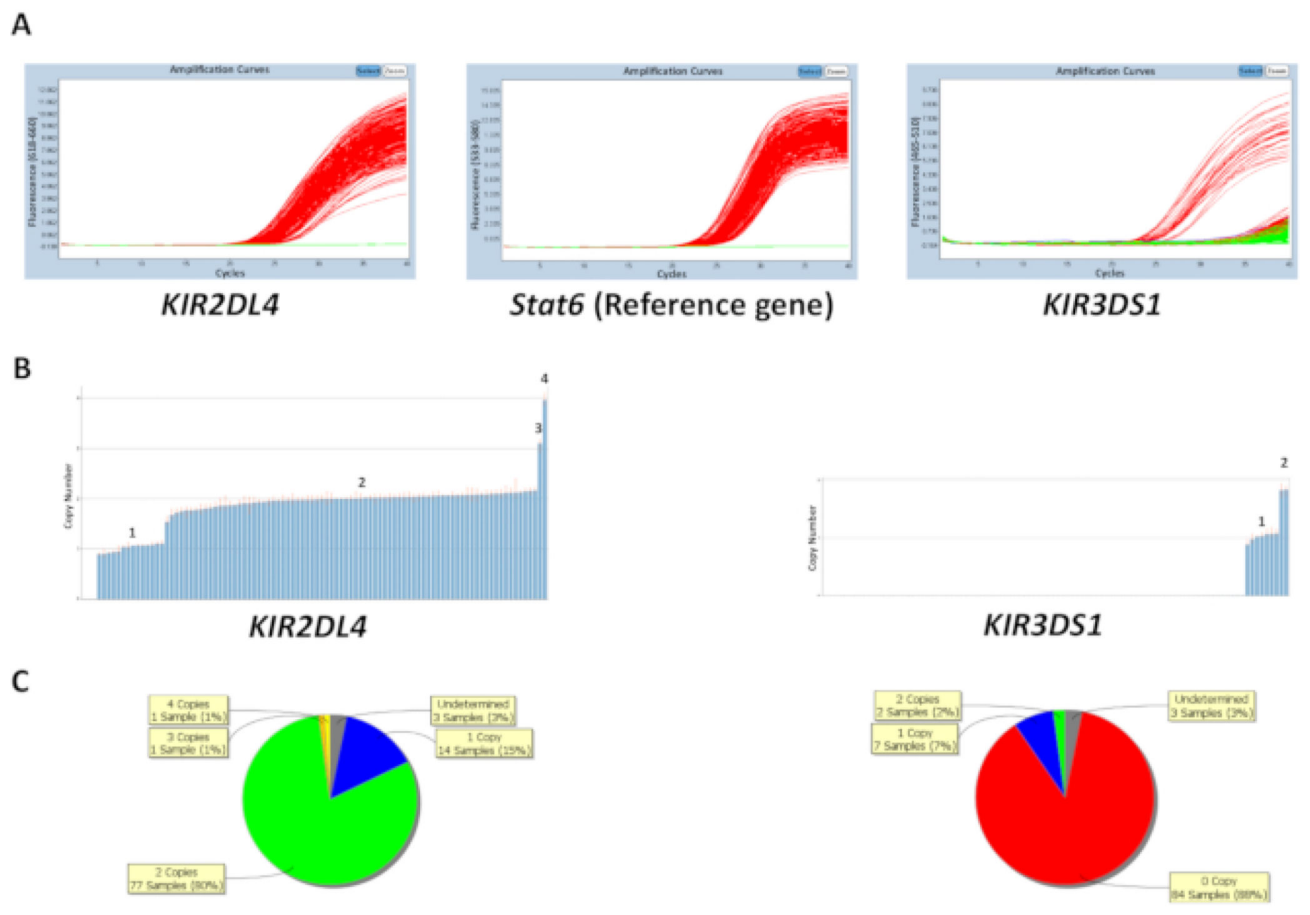
Representative results of a plate for reaction number 5. (**A**) This panel shows amplification plots. (**B**) This panel shows copy number plots. (**C**) This panel shows the copy number distribution. Please click here to view a larger version of this figure.

The copy number can be predicted either based on the known copy number of control DNA samples on the plate or by inputting the most frequent gene copy number (Table 5). Figure 1 shows the results of a plate for a reaction that targets *KIR2DL4* and *KIR3DS1*, as well as the reference gene *STAT6.* The most frequent copy number for *KIR2DL4,* a framework gene in the *KIR* locus, is two copies, whereas the most frequent copy number for *KIR3DS1*, an activating gene, is one copy. The results in the figure show the PCR amplification plots observed on the qPCR software and the copy number data generated from the qPCR data. As shown, the assay is able to distinguish between 0, 1, 2, 3, and 4 *KIR* gene copy numbers. The copy number analysis software also enables a viewing of the distribution of the copy number across the plate as a pie chart or a bar graph. The efficacy of the copy number prediction is lower for samples with a higher copy number.

**Figure 2:**
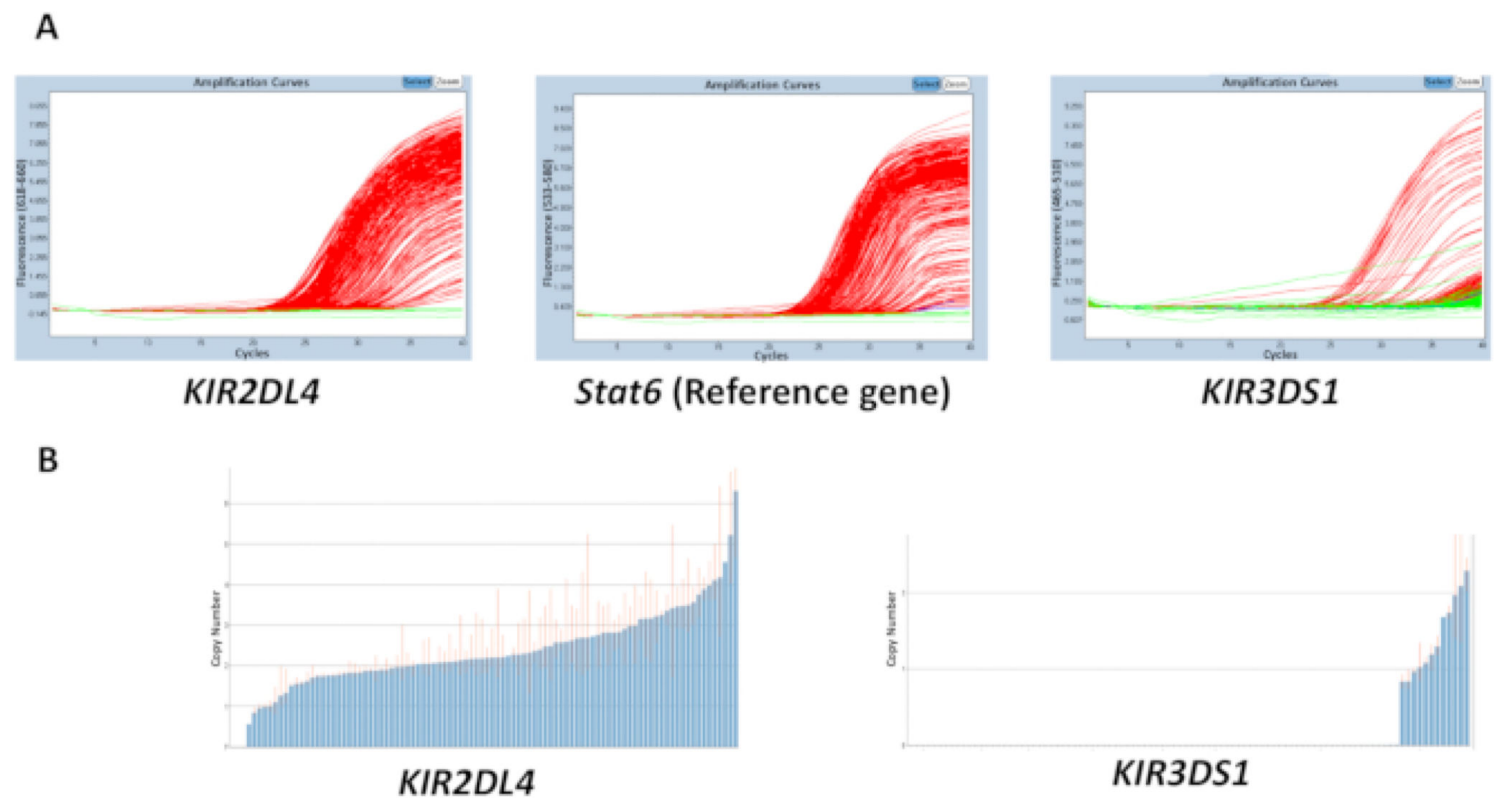
Representative results of a plate with a variable DNA concentration for reaction number 5. (**A**) This panel shows amplification plots. (**B**) This panel shows copy number plots. Please click here to view a larger version of this figure.

The quality of all the materials used in the reactions, gDNA, buffer, primers, and probes, can affect the accuracy of the results obtained. However, discordance in results is most likely to be caused due to variation in the concentration of DNA across a plate. The purity of the extracted gDNA, which can be measured using the 260/280 and 260/230 ratios, can also have an effect on the quality. A 260/280 ratio of 1.8 - 2 and a 260/230 ratio of 2 - 2.2 are desirable. An uneven range of DNA concentrations across a plate can lead to a high variability in the threshold cycle (C_t_) between samples and discordance in the range of the estimated copy number. The results in Figure 2 show the effect the disparity between the C_t_ values across a plate can have on the accuracy in the prediction of the copy number. The red line indicates the range of the estimated copy number for a sample and, ideally, should be as close to an integer as possible.

**Figure 3:**
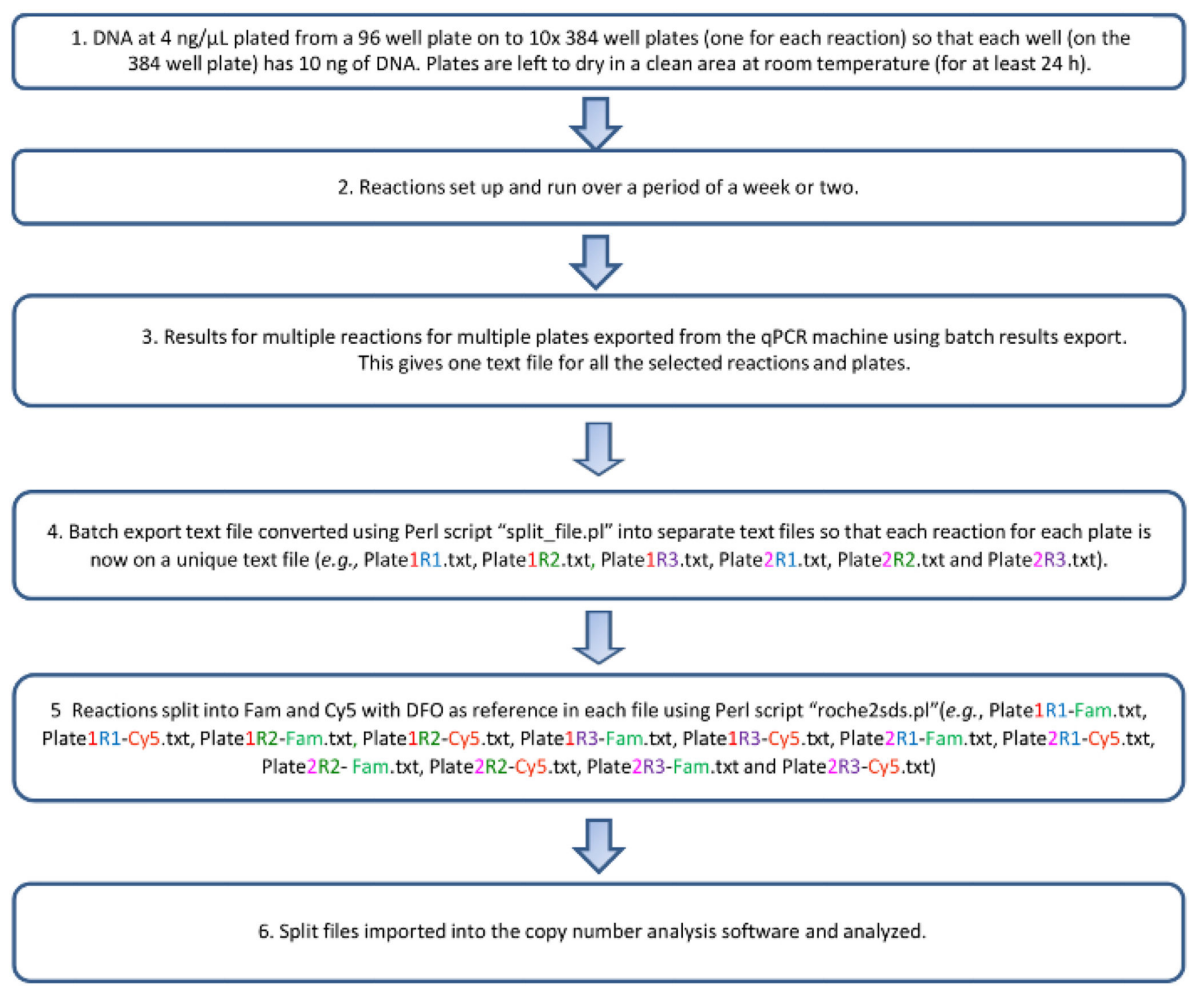
Flowchart of the qKAT protocol. Please click here to view a larger version of this figure.

The copy number data, once analyzed, can be exported as a spreadsheet file in a 96-well format. We used an R script (available on request) to combine the copy number data of all 10 plates that are run as a set into one spreadsheet. Published data about *KIRs* from mostly European-origin populations enables the prediction of LD rules that exist between various genes in the *KIR* complex^1^. These predictions are used to conduct downstream checks on the copy number results obtained (Table 6). Samples that do not conform to the predicted LD between the genes might contain unusual polymorphism or haplotypic structural variations. A flowchart describing the protocol is shown in Figure 3.

A tool called *KIR* Haplotype Identifier (http://www.bioinformatics.cimr.cam.ac.uk/haplotypes/) was developed to facilitate the imputation of haplotypes from the data set. The imputation works on the basis of a list of reference haplotypes observed in a European-origin population^1^. However, the tool also allows for a custom set of reference haplotypes to be used instead. Three separate files are generated; the first file lists all haplotype combinations for a sample, the second file provides a trimmed list of the haplotypes combinations that have the highest combined frequencies, and the third file lists the samples that cannot be assigned haplotypes. Non-assignment of haplotypes could be used as an indicator of novel haplotypes.

## Discussion

We described a novel semi-automated high-throughput method, called qKAT, which facilitates copy number typing of *KIR* genes. The method is an improvement over conventional methods like SSP-PCR, which are low-throughput and can only indicate the presence or absence of these highly polymorphic genes.

The accuracy of the copy number data obtained is dependent on multiple factors, including the quality and concentration-uniformity of the gDNA samples and the quality of the reagents. The quality and accuracy of the gDNA samples across a plate are extremely important since variations in concentration across the plate can result in errors in the calculation of the copy number. Since the assays were validated using European-origin sample sets, data from cohorts from other parts of the world require more thorough checks. This is to ensure that instances of allele dropout or non-specific primer/probe binding are not misinterpreted as copy number variation.

While the assays were designed and optimized to run as high-throughput, they can be modified to run fewer samples. The confidence metric in the copy number analysis software is affected when analyzing fewer samples, but this can be improved if control genomic DNA samples with a known *KIR* gene copy number are included on the plate and additional sample replicates are included.

For laboratories without liquid/plate-handling robots, master mix can be dispensed using multi-channel pipettes and plates can be manually loaded into the qPCR instrument.

The main aim behind the development of qKAT was to create a simple, high-throughput, high-resolution, and cost-effective method to genotype *KIRs* for disease association studies. This was successfully achieved since qKAT has been employed in investigating the role of *KIR* in several large disease association studies, including a range of infectious diseases, autoimmune conditions, and pregnancy disorders^4,24,25,26^.

## Figures and Tables

**Table 1:** Combination and concentration of primers and probes used in each qKAT reaction^27^.

Assay	Genes	Forward Primers	Concentration (nM)	Reverse Primers	Concentration (nM)	Probes	Concentration (nM)
**No 1**	***3DP1***	A4F	250	A5R	250	P4a	150
	***2DL2***	2DL2F4	400	C3R2	600	P5b	150
	***STAT6***	STAT6F	200	STAT6R	200	PSTAT6	150
**No 2**	***2DS2***	A4F	400	A6R	400	P4a	200
	***2DL3***	D1F	400	D1R	400	P9	150
	***STAT6***	STAT6F	200	STAT6R	200	PSTAT6	150
**No 3**	***3DL3***	A8F	500	A8R	500	P4a	150
	***2DS4Del***	2DS4Del	250	2DS4R2	250	P5b	150
	***STAT6***	STAT6F	200	STAT6R	200	PSTAT6	150
**No 4**	***3DL1e4***	B1F	250	B1R	125	P4b	150
	***3DL1e9***	D4F	250	D4R2	500	P9	150
	***STAT6***	STAT6F	200	STAT6R	200	PSTAT6	150
**No 5**	***3DS1***	B2F	250	B1R	250	P4b	150
	***2DL4***	C1F	200	C1R	200	P5b-2DL4	150
	***STAT6***	STAT6F	200	STAT6R	200	PSTAT6	150
**No 6**	***2DL1***	B3F	500	B3R	125	P4b	150
	***2DP1***	D3F	250	D3R	500	P9	150
	***STAT6***	STAT6F	200	STAT6R	200	PSTAT6	150
**No 7**	***2DS1***	B4F	500	B4R	250	P4b	150
	***2DL5***	D2F	500	D2R	500	P9	150
	***STAT6***	STAT6F	200	STAT6R	200	PSTAT6	150
**No 8**	***2DS3***	B5F	250	B5R	250	P4b	150
	***3DL2e9***	D4F	250	D5R	125	P9	150
	***STAT6***	STAT6F	200	STAT6R	200	PSTAT6	150
**No 9**	***3DL2e4***	A1F	200	A1R	200	P4a	150
	***2DS4FL***	2DS4FL	250	2DS4R2	500	P5b	150
	***STAT6***	STAT6F	200	STAT6R	200	PSTAT6	150
**No 10**	***2DS5***	B6F2	200	B6R3	200	P4b	150
	***2DS4***	C5F	250	C5R	250	P5b	150
	***STAT6***	STAT6F	200	STAT6R	200	PSTAT6	150

**Table 2:** Volumes (μL) of 100 μM primer/probe stock solutions to make primer and probe combination aliquots.

Reaction					Primer Aliquots (μL)				Probe Aliquots (μL)		
**R1**	**3DP1**	A4F	A5R	2DL2F4	C3R2	WATER	STAT6F	STAT6R	P4A	P5B	PSTAT6
	**2DL2**	100	100	160	240	200	80	80	60	60	60
**R2**	**2DS2**	A2F	A6R	D1F	D1R	WATER	STAT6F	STAT6R	P4A	P9	PSTAT6
	**2DL3**	160	160	160	160	160	80	80	**80**	60	60
									Note: need 20 μL less water in the MasterMix		
**R3**	**3DL3**	A8F A8FB	A8R	2DS4DELF	2DS4R2	WATER	STAT6F	STAT6R	P4A	P5B	PSTAT6
	**2DS4DEL**	100 100	200	100	100	200	80	80	60	60	60
**R4**	**3DL1E4**	B1F	B1R	D4F	D4R2	WATER	STAT6F	STAT6R	P4B	P9	PSTAT6
	**3DL1E9**	100	50	100	200	350	80	80	60	60	60
**R5**	**3DS1**	B2F	B1R	C1F	C1R	WATER	STAT6F	STAT6R	P4B	P5B-2L4	PSTAT6
	**2DL4**	100	100	80	80	440	80	80	60	60	60
**R6**	**2DL1**	B3F	B3R	D3F	D3R	WATER	STAT6F	STAT6R	P4B	P9	PSTAT6
	**2DP1**	200	50	100	200	250	80	80	60	60	60
**R7**	**2DS1**	B4F	B4R	D2F	D2R	WATER	STAT6F	STAT6R	P4B	P9	PSTAT6
	**2DL5**	200	100	200	200	100	80	80	60	60	60
**R8**	**2DS3**	B5F	B5R	D4F	D5R	WATER	STAT6F	STAT6R	P4B	P9	PSTAT6
	**3DL2E9**	100	100	100	50	450	80	80	60	60	60
**R9**	**3DL2E4**	A1F	A1R	2DS4WTF	2DS4R2	WATER	STAT6F	STAT6R	P4A	P5B	PSTAT6
	**2DS4WT**	80	80	100	200	340	80	80	60	60	60
**R10**	**2DS5**	B6F2	B6R3	C5F	C5R	WATER	STAT6F	STAT6R	P4B	P5B	PSTAT6
	**2DS4TOTAL**	80	80	100	100	440	80	80	60	60	60

**Table 3:** List of probes used in qKAT^1^,^27^. The fluorescent dyes used at the 5' end of the oligo probes P5b, P5b-2DL4, P9, and PSTAT6 were modified to ATTO dyes.

Name	Direction	5´ modification	3´ modification	Sequence (5'→3')	Length	Tm	GC%	Exon	Position
**P4a**	Sense	FAM	BHQ-1	TCATCCTGCAATGTTGGTCAGATGTCA	27	60	44.4	4	425-451
**P4b**	Antisense	FAM	BHQ-1	AACAGAACCGTAGCATCTGTAGGTCCCT	28	62	50	4	576-603
**P5b**	Sense	ATTO647N	BHQ-2	AACATTCCAGGCCGACTTTCCTCTG	25	60	52	5	828-852
**P5b-2DL4**	Sense	ATTO647N	BHQ-2	AACATTCCAGGCCGACTTCCCTCTG	25	61	56	5	828-852
**P9**	Sense	ATTO647N	BHQ-2	CCCTTCTCAGAGGCCCAAGACACC	24	60	62.5	9	1246-1269
**PSTAT6**		ATTO550	BHQ-2	CTGATTCCTCCATGAGCATGCAGCTT	26	62	50		

**Table 4:** Sequences of the primers used in qKAT^1,27^.

Gene	Primers	Direction	Sequence (5´-3´)	Length	Tm	GC%	Exon	Position	Amplicon (bp)	Alleles might be missed
**3DL2e4**	A1F	Forward	GCCCCTGCTGAAATCAGG	18	52	61.1	4	399-416	179	3DL2*008, *021, *027, *038.
	A1R	Reverse	CTGCAAGGACAGGCATCAA	19	53	52.6		559-577		3DL2*048
**3DP1**	A4F	Forward	GTCCCCTGGTGAAATCAGA	19	49	52.6	4	398-416	112	None
	A5R	Reverse	GTGAGGCGCAAAGTGTCA	18	52	55.6		492-509		None
**2DS2**	A2F	Forward	GTCGCCTGGTGAAATCAGA	19	49	52.6	4	398-416	111	None
	A6R	Reverse	TGAGGTGCAAAGTGTCCTTAT	21	51	42.9		488-508		None
**3DL3**	A8Fa	Forward	GTGAAATCGGGAGAGACG	18	50	55.6	4	406-423	139	None
	A8Fb	Forward	GGTGAAATCAGGAGAGACG	19	50	52.6		405-423		3DL3*054, 3DL3*00905.
	A8R	Reverse	AGTTGACCTGGGAACCCG	18	51	61.1		526-543		None
**3DL1e4**	B1F	Forward	CATCGGTCCCATGATGCT	18	51	55.6	4	549-566	85	3DL1*00505, 3DL1*006, 3DL1*054, 3DL1*086, 3DL1*089
	B1R	Reverse	GGGAGCTGACAACTGATAGG	20	52	55		614-633		3DL1*00502
**3DS1**	B2F	Forward	CATCGGTTCCATGATGCG	18	51	55.6	4	549-566	85	3DS1*047; may pick up 3DL1*054.
	B1R	Reverse	GGGAGCTGACAACTGATAGG	20	52	55		614-633		None
**2DL1**	B3F	Forward	TTCTCCATCAGTCGCATGAC	20	52	50	4	544-563	96	2DL1*020, 2DL1*028
	B3R	Reverse	GTCACTGGGAGCTGACAC	18	50	61.1		622-639		2DL1*023, 2DL1*029, 2DL1*030
**2DS1**	B4F	Forward	TCTCCATCAGTCGCATGAA	19	51	47.4	4	545-563	96	2DS1*001
	B4R	Reverse	GGTCACTGGGAGCTGAC	17	49	64.7		624-640		None
**2DS3**	B5F	Forward	CTCCATCGGTCGCATGAG	18	53	61.1	4	546-563	96	None
	B5R	Reverse	GGGTCACTGGGAGCTGAA	18	51	61.1		624-641		None
**2DS5**	B6F2	Forward	AGAGAGGGGACGTTTAACC	19	50	52.6	4	475-493	173	None
	B6R3	Reverse	TCCAGAGGGTCACTGGGC	18	53	66.7		630-647		2DS5*003
**2DL4**	C1F	Forward	GCAGTGCCCAGCATCAAT	18	52	55.6	5	808-825	83	None
	C1R	Reverse	CCGAAGCATCTGTAGGTCT	19	52	52.6		872-890		2DL4*018, 2DL4*019
**2DL2**	2DL2F4	Forward	GAGGTGGAGGCCCATGAAT	19	52	57.9	5	778-796	151	2DL2*009; 782G changed to A.
	C3R2	Reverse	TCGAGTTTGACCACTCGTAT	20	51	45		909-928		None
**2DS4**	C5F	Forward	TCCCTGCAGTGCGCAGC	17	57	70.6	5	803-819	120	None
	C5R	Reverse	TTGACCACTCGTAGGGAGC	19	52	57.9		904-922		2DS4*013
**2DS4Del**	2DS4Del	Forward	CCTTGTCCTGCAGCTCCAT	19	54	57.9	5	750-768	203	None
	2DS4R2	Reverse	TGACGGAAACAAGCAGTGGA	20	53	50		933-952		None
**2DS4FL**	2DS4FL	Forward	CCGGAGCTCCTATGACATG	19	53	57.9	5	744-762	209	None
	2DS4R2	Reverse	TGACGGAAACAAGCAGTGGA	20	53	50		933-952		None
**2DL3**	D1F	Forward	AGACCCTCAGGAGGTGA	17	48	58.8	9	1180-1196	156	None
	D1R	Reverse	CAGGAGACAACTTTGGATCA	20	50	45		1316-1335		2DL3*010, 2DL3*017, 2DL3*01801 and 2DL3*01802
**2DL5**	D2F	Forward	CACTGCGTTTTCACACAGAC	20	52	50	9	1214-1233	120	2DL5B*011 and 2DL5B*020
	D2R	Reverse	GGCAGGAGACAATGATCTT	19	49	47.4		1315-1333		None
**2DP1**	D3F	Forward	CCTCAGGAGGTGACATACGT	20	53	55	9	1184-1203	121	None
	D3R	Reverse	TTGGAAGTTCCGTGTACACT	20	50	45		1285-1304		None
**3DL1e9**	D4F	Forward	CACAGTTGGATCACTGCGT	19	52	52.6	9	1203-1221	93	3DL1*061, 3DL1*068
	D4R2	Reverse	CCGTGTACAAGATGGTATCTGTA	23	53	43.5		1273-1295		3DL1*05901, 3DL1*05902, 3DL1*060, 3DL1*061, 3DL1*064, 3DL1*065, 3DL1*094N, 3DL1*098
**3DL2e9**	D4F	Forward	CACAGTTGGATCACTGCGT	19	52	52.6	9	1203-1221	156	None
	D5R	Reverse	GACCTGACTGTGGTGCTCG	19	54	63.2		1340-1358		None
**STAT6**	STAT6F	Forward	CCAGATGCCTACCATGGTGC	20	54	60			129	
	STAT6R	Reverse	CCATCTGCACAGACCACTCC	20	54	60				

**Table 5:** Most frequent copy number for *KIR* genes commonly observed in European-origin samples.

*KIR* gene	3DL3	2DS2	2DL2	2DL3	2DP1	2DL1	3DP1	2DL4	3DL1 EX9	3DL1 EX9	3DS1	2DL5	2DS3	2DS5	2DS1	2DS4 Total	2DS4 FL	2DS4 DEL	3DL2 ex4	3DL2 EX9
**Most frequent copy number**	2	1	1	2	2	2	2	2	2	2	1	1	1	1	1	2	1	1	2	2

**Table 6:** Linkage disequilibrium between *KIR* genes commonly observed in European-origin populations can be used to check copy number data^1,27^.

	Linkage disequilibrium rules for qKAT based on European populations	Copy number check
**1**	*KIR3DL3, KIR3DP1,KIR2DL4* and *KIR3DL2* are framework genes present on both haplotypes.	*KIR3DL3, KIR3DP1,KIR2DL4* and *KIR3DL2* = 2
**2**	*KIR2DS2* and *KIR2DL2* are in LD with each other	*2DS2*=*2DL2*
**3**	*KIR2DL2* and *KIR2DL3* are alleles of the same gene	*2DL2*+*2DL3*=2
**4**	*KIR2DP1* and *KIR2DL1* are in LD with each other	*2DP1*=*2DL1*
**5**	Exon 4 of *KIR3DL1* and *KIR3DL2* is equal to exon 9 of *KIR3DL1* and *KIR3DL2* respectively.	*3DL1ex4*=*3DL1ex9* AND *3DL2ex4*=*3DL2ex9*
**6**	*KIR3DL1* and *KIR3DS1* are alleles	*3DL1*+*3DS1*=2
**7**	*KIR2DS3* and *KIR2DS5* are in LD with *KIR2DL5*	*2DS3*+*2DS5*=*2DL5*
**8**	*KIR3DS1* and *KIR2DS1* are in LD	*3DS1*=*2DS1*
**9**	Presence of *KIR2DS1* and *KIR2DS4T*otal is mutually exclusive on a haplotype	*2DS1*+*2DS4TOTAL*=2
**10**	*KIR2DS4FL* and *KIR2DS4del* are variants of *KIR2DS4TOTAL*	*2DS4FL*+*2DS4DEL*=*2DS4TOTAL*

## References

[1] Jiang W (2012). Copy number variation leads to considerable diversity for B but not A haplotypes of the human KIR genes encoding NK cell receptors. Genome Research.

[2] Nemat-Gorgani N (2018). Different Selected Mechanisms Attenuated the Inhibitory Interaction of KIR2DL1 with C2^+^ HLA-C in Two Indigenous Human Populations in Southern Africa. The Journal of Immunology.

[3] Norman PJ (2013). Co-evolution of human leukocyte antigen (HLA) class I ligands with killer-cell immunoglobulin-like receptors (KIR) in a genetically diverse population of sub-Saharan Africans. PLoS Genetics.

[4] Nakimuli A (2013). Killer cell immunoglobulin-like receptor (KIR) genes and their HLA-C ligands in a Ugandan population. Immunogenetics.

[5] Bontadini A (2006). Distribution of killer cell immunoglobin-like receptors genes in the Italian Caucasian population. Journal of Translational Medicine.

[6] Graef T (2009). KIR2DS4 is a product of gene conversion with KIR3DL2 that introduced specificity for HLA-A*11 while diminishing avidity for HLA-C. The Journal of Experimental Medicine.

[7] Béziat V, Hilton HG, Norman PJ, Traherne JA (2017). Deciphering the killer-cell immunoglobulin-like receptor system at super-resolution for natural killer and T-cell biology. Immunology.

[8] Blokhuis JH (2017). KIR2DS5 allotypes that recognize the C2 epitope of HLA-C are common among Africans and absent from Europeans. Immunity, Inflammation and Disease.

[9] Martin MP (2002). Epistatic interaction between KIR3DS1 and HLA-B delays the progression to AIDS. Nature Genetics.

[10] Khakoo SI (2004). HLA and NK cell inhibitory receptor genes in resolving hepatitis C virus infection. Science.

[11] van Bergen J (2011). KIR-ligand mismatches are associated with reduced long-term graft survival in HLA-compatible kidney transplantation. American Journal of Transplantation.

[12] Hiby SE (2008). Association of maternal killer - cell immunoglobulin-like receptors and parental HLA - C genotypes with recurrent miscarriage. Human Reproduction.

[13] Nakimuli A (2015). A *KIR B* centromeric region present in Africans but not Europeans protects pregnant women from pre-eclampsia. Proceedings of the National Academy of Sciences.

[14] van Bergen J (2013). HLA reduces killer cell Ig-like receptor expression level and frequency in a humanized mouse model. The Journal of Immunology.

[15] Bachanova V (2016). Donor KIR B Genotype Improves Progression-Free Survival of Non-Hodgkin Lymphoma Patients Receiving Unrelated Donor Transplantation. Biology of Blood and Marrow Transplantation.

[16] Cooley S (2010). Donor selection for natural killer cell receptor genes leads to superior survival after unrelated transplantation for acute myelogenous leukemia. Blood.

[17] Barani S, Khademi B, Ashouri E, Ghaderi A (2018). KIR2DS1, 2DS5, 3DS1 and KIR2DL5 are associated with the risk of head and neck squamous cell carcinoma in Iranians. Human Immunology.

[18] Vilches C, Castaño J, Gómez-Lozano N, Estefanía E (2007). Facilitation of KIR genotyping by a PCR-SSP method that amplifies short DNA fragments. Tissue Antigens.

[19] Ashouri E, Ghaderi A, Reed EF, Rajalingam R (2009). A novel duplex SSP-PCR typing method for KIR gene profiling. Tissue Antigens.

[20] Martin MP, Carrington M (2008). KIR locus polymorphisms: genotyping and disease association analysis. Methods in Molecular Biology.

[21] Crum KA, Logue SE, Curran MD, Middleton D (2000). Development of a PCR-SSOP approach capable of defining the natural killer cell inhibitory receptor (KIR) gene sequence repertoires. Tissue Antigens.

[22] Houtchens KA (2007). High-throughput killer cell immunoglobulin-like receptor genotyping by MALDI-TOF mass spectrometry with discovery of novel alleles. Immunogenetics.

[23] Livak KJ, Schmittgen TD (2001). Analysis of relative gene expression data using real-time quantitative PCR and the 2-ΔΔCT method. Methods.

[24] Traherne JA (2016). KIR haplotypes are associated with late-onset type 1 diabetes in European-American families. Genes and Immunity.

[25] Hydes TJ (2015). The interaction of genetic determinants in the outcome of HCV infection: Evidence for discrete immunological pathways. Tissue Antigens.

[26] Dunphy SE (2015). 2DL1, 2DL2 and 2DL3 all contribute to KIR phenotype variability on human NK cells. Genes and Immunity.

[27] Jiang W (2016). qKAT: A high-throughput qPCR method for KIR gene copy number and haplotype determination. Genome Medicine.

